# *Dianthus superbus* as a critically endangered species in Latvia: evaluation of its growth conditions and conservation possibilities

**DOI:** 10.1093/aobpla/plab051

**Published:** 2021-08-07

**Authors:** Anita Osvalde, Gunta Jakobsone, Ieva Akmane, Andrejs Svilāns, Ilze Dubova

**Affiliations:** 1 Institute of Biology, University of Latvia, 4 O. Vaciesa Street, Riga LV-1004, Latvia; 2 National Botanic Garden, 1 Miera Street, Salaspils LV-2169, Latvia; 3 Botanical Garden of University of Latvia, 2 Kandavas Street, Riga LV-1083, Latvia; 4 Nature Conservation Agency, 7 Baznīcas Street, Sigulda LV-2150, Latvia

**Keywords:** Conservation options, habitat characteristics *in situ*, seed viability and germination, soil agrochemical analysis

## Abstract

*Dianthus superbus* is one of the most endangered species in Latvia and is on the verge of local extinction. Therefore, the aim of this study was to inventory previously identified populations of *D. superbus* in Latvia and to develop activities to conserve this species in accordance with the results obtained *in situ*. Expeditions on 18 previously documented locations, according to the data of Nature Conservation Agency, revealed only three existing *D. superbus* localities in Latvia with a significant number of specimens located in the Latgale region near Silenieki. In 2020, for the first time, two more new *D. superbus* localities were found in the vicinity of these three approved locations. As it is not possible to create micro-reserves in the existing localities which are mainly located either on roadsides or in a cemetery, additional activities are needed to preserve the *D. superbus* in Latvia. *In vitro* culture was initiated from shoot explants obtained *in situ*, from which, in turn, *ex vitro* plantings were done in the National Botanic Garden (NBG) and the Botanical Garden of the University of Latvia (BG UL). Their quality and compliance with plants *in situ* were analysed. Overall, it was concluded that the plants grown *ex situ* were qualitatively equivalent to those obtained *in situ*, as a very high percentage of seed viability and germination was found both for *in situ* and *ex situ* growing *D. superbus*. Based on the results obtained we could conclude that *D. superbus* is a species that is able to adapt to different soils with a wide range of nutrient levels, moisture conditions and pH, as long as there are three main preconditions—adequate lighting, low overgrowth of other plant species and non-destructive human activities.

## Teaser

*Ex situ* conservation of the critically endangered species *Dianthus superbus* in Latvia outside natural habitats was carried out by introducing and maintaining *in vitro* collections and field plantings in the botanical gardens. As *D. superbus* is able to adapt to a wide range of soil conditions in terms of nutrient and organic matter content and soil reaction, the conservation of this species can also be implemented *inter situs* in urban areas and dendrological parks. There are three main preconditions for the successful growth of *D. superbus* in Latvia—adequate lighting, low overgrowth of other plant species and non-destructive human activity.

## Introduction

*Dianthus superbus* is widely but patchily distributed in Europe and northern Asia. Although not considered to fall within a threatened category in Europe as a whole, *D. superbus* is mentioned as a seriously threatened species in several European countries. It is considered to be possibly extinct in the Netherlands, critically endangered in the Czech Republic and Romania, vulnerable in France, Sweden and Poland, near threatened in Hungary and rare in Denmark (Mikulík and Vinter 2001–2002; Holobiuc *et al.* 2004–2005; Rosenthal 2010; Kostrakiewicz-Gierałt 2013; Hardion *et al.* 2018). *Dianthus superbus* is protected in the south of Finland, and a variant growing on the serpentines is critically endangered (Lehmuskallio 2014).

In the Baltic States *D. superbus* is included in the category of endangered species in Red Data Book of Lithuania (Rašomavičius 2007) and it is classified as vulnerable species in Estonia (https://elurikkus.ee/bie-hub/species/4309#redlist). In Latvia *D. superbus* as an endangered species on the edge of extinction is included in the Regulations of the Cabinet of Minister of Latvia No. 396 (Anonymous 2000) and the first category of threat in Red Data Book of Latvia (Kabucis 2003). *Dianthus superbus* is also included in the Red Data Book of the Baltic Region (Ingelög *et al.* 1993).

The decline of suitable habitats is considered to be a major threat for the species (Miranto *et al.* 2012; Allen *et al.* 2014). Due to changes in land use and agricultural practices, the natural or semi-natural habitats of *D. superbus* were intensively farmed or practically abandoned in the last century in Europe, resulting in highly fragmented or extinct habitats. Spatial isolation can lead to low genetic diversity, weakened fitness and declining population size, leading to a high risk of local extinction (Kostrakiewicz-Gieralt 2013; Hardion *et al.* 2018).

Given the progressive processes of extinction of many populations and the lack of data on variations in population characteristics, it should be emphasized that further demographic research is still needed. Demographic data can provide information not only on the status of population but also on its survival prospects *in situ* and serve as a basis for appropriate conservation programmes.

Seed germination is a significant phase in the reproductive cycle of species. An appropriate temperature, enough water and oxygen are very important factors for achieving a high germination rate. According to Mikulík and Vinter (2001–2002), the parameters of *D. superbus* germination were very high immediately after the maturation of capsules, exceeding 80 %. At the same time, seeds of *D. superbus* are very susceptible to loss of germination ability. Thus, after 5 years of storage, germination energy and rate decreased twice (Kurek *et al.* 2019). Oxidative damage, deterioration of nucleic acids and fall of enzymatic activity are mentioned as potential drivers of the seed aging process (Kurek *et al.* 2019). In general, seed germination takes place over a certain temperature range, the most important of which is related to the phytogenetic adaptation of the species. An optimum temperature for *D. superbus* germination between 15 and 25 °C was reported by Hiromasa and Noboru (2006). Studies in the Czech Republic also showed a high germination rate of *D. superbus* at 20 °C (Šebánek 1992). Such a temperature from the germination point of view is very characteristic of many plant species also in the temperate and boreal zone.

One of the important factors for species growth and persistence is appropriate soil conditions. Only general soil characteristics for *D. superbus* are available. *Dianthus superbus* is considered a species favouring rich, well-drained sandy or loamy soil with neutral or slightly alkaline pH (Nature Gate 2021). Soils with a high content of organic matter in the upper horizons and a strong hydromorphic character have also been reported as appropriate for *D. superbus* (Gavrilova 1999; Hardion *et al.* 2018). Therefore, more detailed studies on soil properties and nutrient status are necessary not only to understand the ecology and the conservation potential of *D. superbus* in the wild, but also for preservation *ex situ* and reintroduction needs.

Although endangered plant species in Latvia are relatively well documented, information on *D. superbus* is mainly available from herbarium materials, literature and database of the Nature Conservation Agency. As mentioned by Prieditis (2014)*D. superbus* is one of the most endangered plants in Latvia and is on the verge of extinction. There are very few data on changes in the characteristics of *D. superbus* population in recent decades and the current status of this species in Latvia (Jakobsone *et al.* 2020). So far, no research has been carried out on the possibilities of conservation of this species in Latvia. Therefore, the aim of this study was to inventory previously identified populations of *D. superbus* in Latvia in order to develop the activities for the conservation of this species in accordance with the results obtained *in situ*. To achieve this aim, a number of tasks were set: (i) to explore habitat characteristics of the *D. superbus in situ*, (ii) to determine soil agrochemical composition *in situ* and *ex situ*, (iii) to compare reproduction characteristics and potential *in situ* and *ex situ*, (iv) to reveal species conservation options.

### The study species

Superb Pink *D. superbus* (Caryophyllaceae) is a loosely tufted perennial, medium-sized (35–80 cm) (Gavrilova 1999), clonal forb with a strong primary root and an ascending or creeping rhizome. The vegetative stems are topped with oblong leaves, while generative shoots bear a solitary flower (Ø 3–5 cm) (Priedītis 2014) or 3–15 flowers grouped in inflorescences (Kabucis 2003). Typical inflorescences have one primary flower, secondary flowers on each side of the central axis and tertiary flowers on each side of the lateral axis. The flowers are composed of five free, deeply cut fringed petals forming a functional tube enclosed by the tubular calyx, are whitish in colour, have a long calyx tube, up to 2.5 cm long, and are strongly scented, especially during the night, indicating pollination by night-active flower visitors. However, the flowers do not exclude diurnal flower visitors (Jürgens *et al.* 2003).

*Dianthus superbus* is known to form subspecies, some of which have a limited geographical range. As in most species range, the subspecies found in Latvia is attributed to *D. superbus* subsp. *superbus* (https://www.latvijasdaba.lv/augi/dianthus-superbus-l/). To the east of Latvia, in Russia, and in Ukraine, another subspecies is common—*D. superbus* subsp. *stenocalyx* (Jalas and Suominen 1988). Recent studies have shown that the taxonomy of this species in Europe is still unresolved, calling into question the subsp. *sylvestris*, while supporting subsp. *alpestris* (Hardion *et al.* 2020).

The ecotope for *D. superbus* is wet meadows and fens (Gavrilova 1999); individuals form small groups in fens, periodically wet meadows on peat or clay on carbonic deposits (Priedītis 2014). In contrast to these findings, habitats for *D. superbus* in Finland are sandy and gravelly riverbanks, shore meadows, rocky embankments, dry commons and roadsides (Lehmuskallio 2014). In Greece, it is also found in grassy clearings (Lafranchis and Sfkar 2009).

## Methods

### Inspection *in situ*

**Figure 1.**
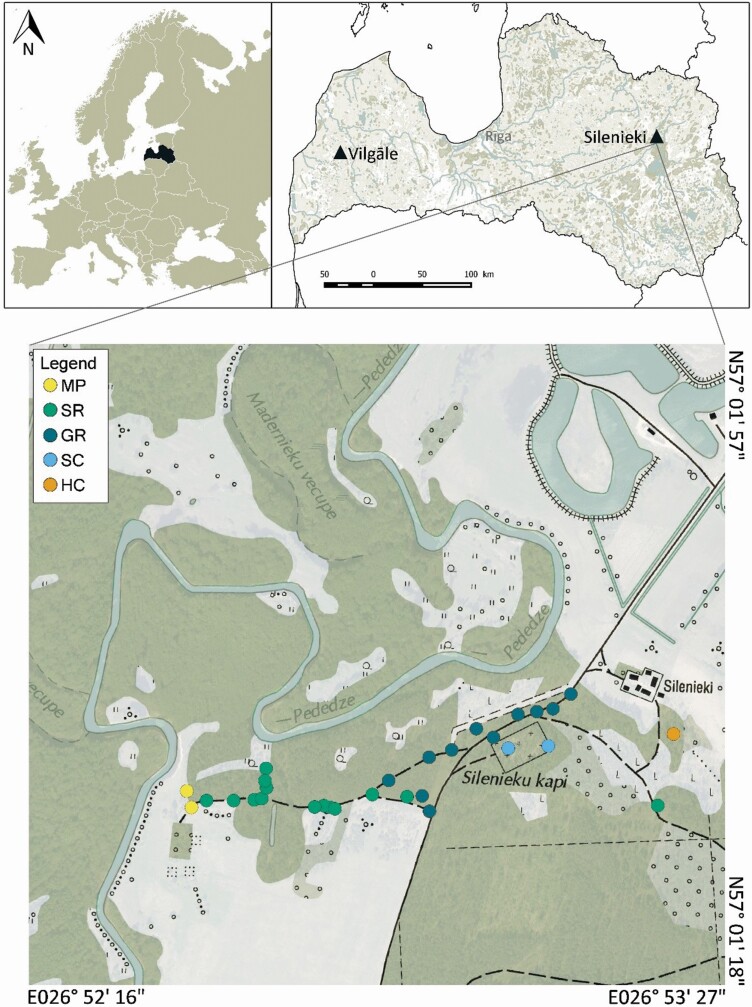
Location of *Dianthus superbus* research area. The map on top on the left shows the position of Latvia within the Europe. The map on top on the right illustrates the two locations of *D. superbus* in Latvia (black triangles). The map below shows the localities of *D. superbus* in Silenieki area (coloured circles). Square size is 1.2 × 1.2 km. MP—meadow between Pededze shore and forest edge; SR—sand road and at the side of the road in pine forest near the village Silenieki; GR—gravel road edge near the village Silenieki; SC—Silenieki-cemetery; HC—hillock with clearing (Latvian Geospatial Information Agency, topographic map, M1:10 000, 2010; SIA Envirotech, ESRI File geodatabase, GIS Latvija 10.2, M1:500 000, 2016).

In 2017, numerous expeditions were carried out to verify the data on all previously known locations of *D. superbus* (Jakobsone *et al.* 2020). Unfortunately, in most of them *D. superbus* was no longer discovered. This study was continued and existing localities were re-surveyed in 2019 and 2020. During the expedition in 2020, *D. superbus* was additionally searched in Silenieki (Fig. 1), surveying a wider area near the only locality in Latvia with substantial population size. In 2020, *D. superbus* was found for the first time in a meadow between the River Pededze and a forest at the end of a sand road (MP) and on a hillock with clearing at the farmstead ‘Priežulejas’ (HC) (Fig. 1). A description of vegetation with an inventory of the accompanying species was done for the localities where *D. superbus* specimens were found. All generative shoots and the number of flowers on them were counted. Seeds were collected for seed viability and germination tests, as well as for an *ex situ* collection.

### Preservation *ex situ*

In order to survive, parallel conservation measures are needed for the endangered species: *in situ* and *ex situ*, including *in vitro*. *Ex situ* activities were performed in the National Botanic Garden (NBG) and in the Botanical Garden of the University of Latvia (BG UL). *In vitro* culture of *D*. *superbus* was developed in the Department of Eco-Physiology of NBG with cuttings collected in 2017 at the Silenieki findings. The propagation procedure is given below. *In vitro* obtained plantlets were used to establish plantations in both botanical gardens in the spring of 2018.

### Seed viability and germination

To evaluate the existing *in situ* populations of *D. superbus*, tests were carried out on seed viability and germination. Seeds were collected in populations near Silenieki (GR, SR, MP) and in NBG and BG UL plantations, established under conditions close to natural. The embryo viability was determined using 1 % 2,3,5-triphenyl-2H-tetrazolium chloride (TTC) test (modified Ramsey and Dixon 2003). One part of the seeds collected *in situ* in 2017 was analysed directly after collection, the other—in the following spring. For this part dry weight of 100 seeds was determined, the seeds were stored in a refrigerator at 4 °C during the winter and the viability of the seeds was tested in the spring of 2018. For TTC test, 50 seeds were analysed in three repetitions. For germination test, 30 seeds were put in Petri plates on double-layer filter paper, one on top, moistened with distilled water. The same experiments were repeated with seeds collected in autumn 2019 with seeds collected *in situ* and from *in vitro* obtained plants in both botanical gardens. Germination test was done in an artificial climate camera with a 16/8 h light/dark photoperiod and temperature regime 23 °C/15 °C. Seeds were counted every 3 days. The TTC test was repeated in 2020 after additional sites with *D. superbus* (HC, MP) were found.

### Soil analyses

Soil samples were collected several times in the period from 2017 to 2020 from the populations near Silenieki (GR, SR), in 2020 from the newly discovered populations (MC, HC) and in 2019–20 from the *ex situ* plantations in NBG and BG UL. Samples were taken from the plant root zone at 0–20 cm depth, dried to air-dry condition and sieved through a 2-mm sieve. The plant-available concentrations of nutrients (N, P, K, Ca, Mg, S, Fe, Mn, Zn, Cu, Mo and B) in soil samples were extracted with 1 M HCl solution, in which the soil–extractant volume mixture ratio was 1:5 (Rinkis *et al.* 1987). The soil pH was detected in 1 M KCl (soil–extractant mixture 1:2.5), and electrical conductivity (EC) was measured by using distilled water extract (soil/distilled water ratio 1:5). The levels of K, Ca, Mg, Fe, Cu, Zn and Mn were estimated by microwave plasma atomic emission spectrometer (MP-AES) Agilent 4200 (Anonymous 2021), those of N, P, Mo and B were analysed by the colourimetry, S by turbidimetry (spectrophotometer JENWAY 6300). Soil pH was determined potentiometrically by pH-meter Sartorius PB-20, EC with the conductometer Hanna EC 215, soil organic matter content according to Tjurin method (Rinkis *et al.* 1987).

### In vitro

*In vitro* culture initiation was done with cuttings from plants obtained near the Silenieki at the edge of a gravel road. Sterilization procedure: cuttings with two internodes were soaked in weak K permanganate solution overnight, then rinsed in distilled water and immersed in ½ ACE (commercial solution with active Cl^−^) for 15 min. After the sterilization procedure, the cuttings were rinsed four times in sterile distilled water and placed in culture tubes. For the initiation of *in vitro* culture, 16 × 100 mm Pyrex culture tubes (SIA MyLab, Latvia, Riga) were used with 14 mL medium and closed with foil. Hereafter the cultivation was made in 24 × 200 mm Duran culture tubes (‘Antoniadis Electronics Educational Ltd’, Cyprus) with 25 mL medium, closed with foil; in each tube—three explants. The cultivation media based on Murashige and Skoog (1962) inorganic substances. The list of organic substances was modified in the Department of Eco-Physiology, NBG: the culture initiation medium contains 0.12 mg L^−1^ 6-benzylaminopurine, 0.03 mg L^−1^ α-naphtalene acetic acid and β-indole acetic acid, whereas the cultivation medium was used without phytohormones. The reagents for *in vitro* cultivation were obtained from Duchefa Biochemie B.V., Haarlem, the Netherlands. The pH of the culture media was adjusted to 5.8 using either HCl or NaOH (both reagents from Sigma-Aldrich®, St Louis, MO, USA) before gelling with agar (Duchefa Biochemie B.V.) prior to autoclaving for 15 min at 121 °C and 1.05 kg cm^−2^. There were 15 2.5 × 15 cm culture tubes per treatment closed with polypropylene cups (Sigma-Aldrich®).

Culture tubes with explants were placed in a cultivation chamber under master TL-D Super 80 58W/840 white luminescent lamps (Philips, Pila, Poland), with a light intensity of 27.6 µmol m^−2^ s^−1^ and a 16-h photoperiod for 2 × 2 months, i.e. transplanting *in vitro* was done every 2 months.

For slow growth conditions, the plantlets were periodically held in a cultivation chamber under master TL-D Super 80 58W/840 white luminescent lamps (Philips), with a light intensity of 27.6 µmol m^−2^ s^−1^ and a 12-h photoperiod and 4–6 for 8 months.

Transplanting *in vitro* was done every 2 months.

### *Ex vitro* in outdoor conditions

The cuttings from *in vitro* obtained plantlets were replanted in substrate in *ex vitro* conditions using plant boxes with neutral fertilized peat (pH 6–7) on March 2018. The plant boxes were placed under white luminescent lamps for 2 months. The rooted plants were then transplanted outdoors in the territory of NBG and BG UL, In NBG, the plants were planted in two places: in a perennial plant bed on 5 m^2^ plot and on the southern slope of the pond, in semi-natural conditions on four plots (1.2 × 1.2 m). In BG UL, *in vitro* derived plants were transplanted (i) in ornamental, (ii) in semi-natural conditions on the shore of a pond with *Taxus baccata* and *Tussilago farfara* (2 × 2 m) and (iii) in semi-natural conditions on the shore of a pond with *Matteuccia struthiopteris* (2 × 2 m). The first observations were made in 2019 during flowering; they were repeated in 2020. From the beginning of flowering, plants were evaluated by counting the generative shoots and the number of flowers per shoot.

Statistical analysis of the results was done using MS Excel 2016. Standard errors (SEs) were calculated to reflect the mean results of seed viability and germination rate, as well as the number of flowers per generative shoot. Heterogeneity of soil nutrient concentrations was characterized by the coefficient of variation (CV). The Student’s *t*-test ‘Two-Sample Assuming Unequal Variances’ (*P* < 0.05) was used to check the significance of the differences between *D. superbus* sites *in situ* and *ex situ* in the number of flowers per generative shoot and seed quality parameters.

## Results

### In situ

**Figure 2.**
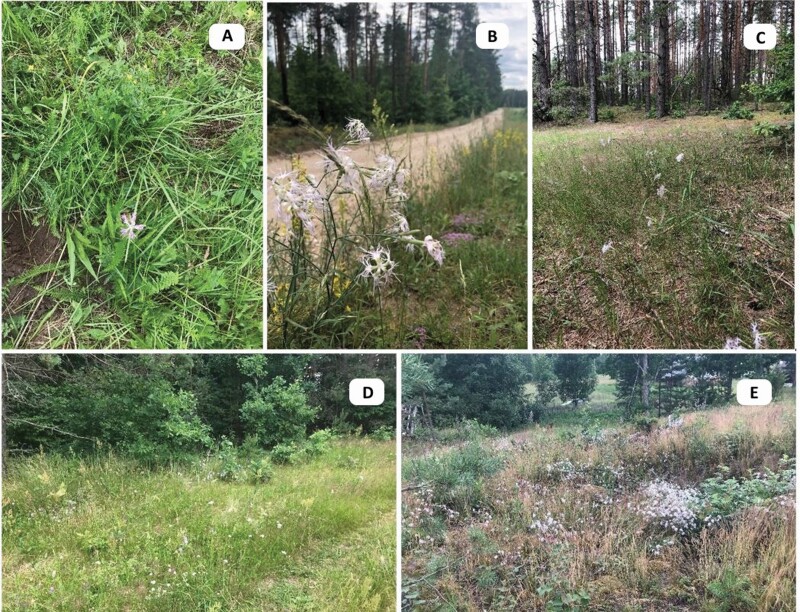
*Dianthus superbus in situ*: (A) in the micro-reserve Dzirnieki at the lake Vilgāle (DZ); (B) at the gravel road edge near the village Silenieki (GR); (C) on the sand road and at the side of the road in pine forest near the village Silenieki (SR); (D) on the edge of the forest by the Pededze River (MP); (E) on the hillock with clearing (HC) (photo: Gunta Jakobsone).

Examination of 18 previously dated localities revealed that *D. superbus* had survived only in four: one in Kurzeme region—in the micro-reserve Dzirnieki at Lake Vilgāle (DZ) and in Latgale region—locality near Lake Lubāns: in the Silenieki-cemetery (SC), at a gravel road edge near the village Silenieki (GR), on a sand road and on the side of the road in a pine forest near the village Silenieki (SR) (Fig. 1). Overgrowth with other tall herbaceous species or shrubs was found in areas where carnations were no longer observed. Cattle grazing and trampling were also found at some previously dated *D. superbus* sites. In the only micro-reserve for *D. superbus* in Latvia (DZ), which is located in a private territory, the meadow was mowed early and low. In 2017, only one generative shoot with one flower and two buds was found in August (Fig. 2A). During the expedition in 2020, *D. superbus* was first found in two new locations near Silenieki: in a meadow between the Pededze River and the forest at the end of the sand road (MP), as well as on the hillock with a clearing at the farmstead ‘Priežulejas’ (HC) (Fig. 1).

*Dianthus superbus* was growing in cemetery, located on a sandy pine forest hill (SC), on gravel road edge in clearing (GR); near a pine forest with some other tree species on sandy, dry roadsides (SR); in a meadow between the shore of the Pededze River and the edge of a forest at the end of a sand road (MP); and on the hillock with ~2 years old clearing at the farmstead ‘Priežulejas’ (HC). The study showed that the accompanying plant species in these localities form a low and sparse ground cover (Fig. 2B–E). Detailed characteristics of the *D. superbus* habitats *in situ* are given in Table 1, characteristics of the environment of *D. superbus* in semi-natural conditions and as ornamental plant *ex situ*—in Table 2. Significant differences in soil reaction were found for five sites where *D. superbus* was currently distributed *in situ*: pH_KCl_ from 3.75 to 6.96. The substrate reaction from acidic to neutral was also found for *D. superbus ex vitro* in NBG and BG UL (3.66–7.14) (Table 2).

**Table 1.** Characteristics of the *Dianthus superbus* habitats *in situ.*

Site symbol	Deposit	Habitat	Accompanying species (main—in bold)	pH/_KCl_
DZ	Micro-reserve Dzirnieki at Lake Vilgāle	EU grassland habitat 6410_4, variant with inclusions of 6270*_1	***Achillea millefolium***, ***Deschampsia flexuosa***, ***Filipendula vulgaris***, ***Galium boreale*, *Pimpinella saxifraga*, *Plantago lanceolata*,***Agrostis stolonifera*, *Alchemilla vulgaris s.l.*, *Angelica sylvestris*, *Anthoxanthum odoratum*, *Anthriscus sylvestris*; *Campanula patula*, *C. rotundifolia*, *Centaurea jacea*, *Cirsium arvense*, *C. oleraceum*, *Lychnis flos-cuculi*, *Dactylis glomerata*, *Dianthus deltoides*, *Festuca ovina s.str.*, *Filipendula ulmaria*, *G. verum*, *Geranium palustre*, *Holcus lanatus*, *Hypericum maculatum*, *Knautia arvensis*, *Lathyrus pratensis*, *Leontodon autumnalis*, *Leucanthemum vulgare*, *Molinia caerulea*, *Nardus stricta*, *Phleum pratense*, *Plantago media*, *Polygala vulgaris*, *Polygonum bistorta*, *Potentilla erecta*, *Ranunculus acris*, *Rumex acetosa*, *Sieglingia decumbens*, *Stellaria graminea*, *Taraxacum officinale s.l.*, *Tragopogon pratensis*, *Trifolium dubium*, *T. montanum*, *T. pratense*, *Trollius europaeus*, *Veronica chamaedrys*, *Vicia cracca*, *Viola montana*, *Equisetum arvense*	4.38
SC	Silenieki-cemetery	Cemetery	***Pinus sylvestris*, *Thymus serphyllum*, *Dianthus barbatus*, *Agrostis stolonifera***, *Antennaria dioica*, *Artemisia campestris*, *Erigeron acris*, *E. canadensis*, *Festuca rubra s.l.*, *Hieracium pilosella s.l.*, *H. umbellatum*, *Jasione montana*, *Leontodon autumnalis*, *Leucanthemum vulgare*, *Potentilla argentea*, *Rumex acetosa*, *R. acetosella*, *Scleranthus annuus*, *Sedum acre*, *Sieglingia decumbens*, *Trifolium repens*	
GR	Gravel road edge near the village Silenieki	Road edge	***Pinus sylvestris*, *Juniperus communis*, *Thymus serphyllum*, *Vaccinium myrtillus*, *V. vitis-idaea*, *Agrostis stolonifera*, *Dianthus arenarius*, *Lycopodium clavatum*, *Dicranum polysetum*, *Pleurozium schreberi*, *Ptilium crista-castrensis***, *Amelanchier spicata*, *Calluna vulgaris*, *Frangula alnus*, *Padus avium*, *Populus tremula*, *Quercus robur*, *Antennaria dioica*, *Anthoxanthum odoratum*, *Artemisia campestris*, *A. vulgaris*, *Calamagrostis epigeios*, *Carex ericetorum*, *Centaurea jacea*, *Dactylis glomerata*, *Festuca ovina s.str.*, *Galeopsis ladanum*, *Hieracium pilosella s.l.*, *H. umbellatum*, *Jasione montana*, *Knautia arvensis*, *Koeleria glauca*, *Leontodon autumnalis*, *Linaria vulgaris*, *Melampyrum pratense*, *Phleum nodosum*, *Pimpinella saxifraga*, *Polygala vulgaris*, *Polygonatum odoratum*, *Rumex acetosa*, *R. acetosella*, *Scleranthus annuus*, *Sieglingia decumbens*, *Silene nutans*, *Solidago virgaurea*, *Stellaria graminea*, *Trifolium aureum*, *Veronica chamaedrys*, *V. spicata*, *Vicia cracca*, *Viola montana*, *Dryopteris carthusiana*	6.96
SR	Sand road and at the roadside in a pine forest near the village Silenieki	Road edge	***Pinus sylvestris*, *Thymus serphyllum*, *Achillea millefolium*, *Dianthus arenarius*, *D. deltoides*, *Lycopodium clavatum*, *Dicranum polysetum*, *Pleurozium schreberi*, *Ptilium crista-castrensis***, *Frangula alnus*, *Padus avium*, *Quercus robur*, *Rubus caesius*, *Achillea millefolium*, *Anthoxanthum odoratum*, *Artemisia campestris*, *Berteroa incana*, *Calamagrostis epigeios*, *Hieracium pilosella s.l.*, *H. umbellatum*, *Jasione montana*, *Knautia arvensis*, *Mycelis muralis*, *Pimpinella saxifraga*, *Plantago media*, *Polygonatum odoratum*, *Rumex acetosa*, *R. acetosella*, *Silene nutans*, *Veronica spicata*	4.52
MP	Meadow between Pededze shore and forest edge at the end of the road	Semi-natural dry grassland	***Agrostis tenuis*, *Festuca pratensis*, *Galium album*, *Knautia arvensis*, *Achillea millefolium*,***Stellaria graminea*, *Dianthus deltoides*, *Veronica chamaedrys*, *Rumex acetosella*, *Thymus serphyllum*, *Artemisia campestris*	5.27
HC	On the hillock with ~2 years old clearing at the farmstead ‘Priežulejas’	Pine forest clearing	***Pinus sylvestris*, *Populus tremula*, *Sorbus aucuparia*, *Quercus robur*, *Frangula alnus*, *Festuca ovina s.str.*, *Vaccinium vitis-idaea***, *Convallaria majalis*, *Rumex acetosella*, *Calamagrostis epigeios*, *Agrostis tenuis*	3.75

**Table 2.** Characteristics of the environment of *Dianthus superbus* in semi-natural conditions and as ornamental plant *ex situ* in National Botanic Garden (NBG) and in the Botanic Garden of the University of Latvia (BG UL).

Botanic garden	Planting place	Substrate characteristic	pH/_KCl_
NBG	Pond south slope, semi-natural conditions	Dry sandy soil	6.25–6.51
NBG	Perennial-bed	Peat	3.66
BG UL	Bed for threatened plants	Dry cultivated soil	7.14
BG UL	Pond slope	Dry sandy soil	6.81–6.9
BG UL	Pond slope	Dry sandy soil	6.88–6.9

### Soil analyses

In general, wide ranges of nutrient concentrations, soil reaction and organic matter content were found at various *D. superbus* sites in Latvia (Table 3). Of the macronutrients, the highest CV was determined for Ca (216 %) and Mg (259 %), the lowest for S (44 %). For micronutrients, the highest concentration variability was determined for Fe (81 %) and Cu (68 %), the lowest for Mo (28 %). Since the EC characterize the level of soluble ions in the soil, the CV for EC (59 %) coincided with the average of N, K, S and Na.

**Table 3.** Nutrient concentration (mg L^−1^, 1 M HCl extraction), soil pH and EC and organic matter content in air-dry soils from *Dianthus superbus* in situ sites in Latvia, 2017–20. *Coefficient of variation.

	Micro-reserve Dzirnieki at the Lake Vilgāle (2017)	Gravel road edge near the village Silenieki/1 (2017–20)	Gravel road edge near the village Silenieki/2 (2017)	Sand road and at the side of the road in pine forest near the village Silenieki (2017–20)	Meadow between Pededze shore and forest edge (2020)	Hillock with clearing (2020)	Range	CV*, %
N	113	8–33	25	28–55	23	10	8–113	83.5
P	256	150–213	213	119–610	278	116	116–610	61.5
K	165	77–180	175	65–327	80	63	63–327	61.5
Ca	770	375–409	14 250	275–690	1043	778	275–14 250	216.2
Mg	120	50–57	5000	47–80	208	94	47–5000	258.4
S	13	4–7	16	6–11	7	6	4–16	43.7
Fe	1840	221–270	630	524–995	399	262	221–1840	81.4
Mn	190	65–65	100	95–120	91	51	51–190	41.8
Zn	7.5	3.5–3.6	6.0	3.5–8.0	6.0	10.5	3.5–10.5	41.9
Cu	2.50	0.50–0.50	1.85	0.70–0.75	0.90	0.65	0.50–2.50	68.3
Mo	0.03	0.03–0.04	0.03	0.03–0.05	0.04	0.02	0.02–0.05	27.6
B	0.2	0.2–0.3	0.2	0.1–0.6	0.3	0.3	0.1–0.6	53.0
Na	9.0	4.9–12.0	18.5	6.0–21.0	10.5	8.9	4.9–21.0	49.0
pH_KCl_	4.38	4.22–4.59	6.96	4.13–4.56	5.27	3.75	3.75–6.96	19.9
EC_H2O_ mS/cm	0.24	0.12–0.19	0.56	0.12–0.2	0.25	0.22	0.12–0.56	59.2
Organic matter, %	4.15	3.40–4.00	5.70	2.85–7.50	4.15	8.55	2.85–8.55	38.2
Bulk density, g cm^3^	1.09	1.03–1.11	1.07	0.93–1.19	1.07	0.90	0.9–1.19	9.3

According to soil analyses, *D. superbus* localities in Latvia were mainly found on mineral soils with an organic matter content of 2.85–8.55 %.

In general, soil pH and EC, as well as nutrient (N, P, K, Ca, S, Fe, Mn and Mo) concentrations for *ex situ* cultivation of *D. superbus* in NBG and BG UL were within the range found in the wild (Tables 3 and 4). The maximum concentrations of Ca, Zn, Cu, B and Na found in soils of botanic gardens were higher. While in semi-natural growing sites *ex situ* the soil organic matter content ranged from 4.0 to 7.5 %, there was also one site developed on peat soil (perennial plant bed NBG) with an organic matter content of 39 %.

**Table 4.** Nutrient concentration (mg L^−1^, 1 M HCl extraction), soil pH and EC and organic matter content in air-dry soils from *Dianthus superbus ex situ* sites in National Botanic Garden (NBG) and Botanic Garden of the University of Latvia (GB UL), 2019–20. *Coefficient of variation.

	NBG		BG UL			Range	CV*, %
	Semi-natural meadow (2019–20)	Ornamental plant bed (2019)	Bed for threatened plants (2019–20)	Shore of central pond (2019–20)	Shore of fern pond (2019–20)		
N	68–88	40	28–93	40–89	33–65	28–93	42.8
P	192–234	73	534–556	338–360	256–256	73–556	50.3
K	122–183	124	125–139	141–196	115–142	115–196	19.7
Ca	2412–2700	1045	11 513–11 700	4400–4898	4450–4513	1045–11 700	71.7
Mg	373–550	325	6067–6500	1753–1800	1538–1600	325–6500	103.1
S	14–15	4	18–24	16–18	15–16	4–24	33.7
Fe	895–1099	245	1326–1740	849–900	475–495	245–1740	51.7
Mn	145–145	16.5	116–125	63–65	88–110	16.5–145	44.1
Zn	1.80–3.4	4.8	53–60	17.5–18	16.5–19.5	1.8–60	97.2
Cu	2.00–2.25	0.70	9.45–10.5	4.65–5.1	4.50–4.70	0.7–10.5	67.1
Mo	0.04–0.04	0.025	0.05–0.05	0.04–0.07	0.04–0.04	0.025–0.07	27.8
B	0.5–0.5	0.3	0.2–0.5	0.7–0.9	0.5–0.8	0.2–0.9	41.2
Na	10.9–22.0	25.0	43.0	37.5	20.0	10.9–43.0	44.9
pH_KCl_	6.25–6.51	3.66	7.14–7.14	6.81–6.90	6.88–6.90	3.66–7.14	16.9
EC_H2O_ mS/cm	0.38–0.44	0.21	0.27–0.33	0.40–0.41	0.39–0.41	0.21–0.44	21.0
Organic matter, %	4.00–5.70	38.68	4.80–5.50	5.20–7.50	6.20–6.70	4.00–38.68	117.9
Bulk density, g cm^3^	1.16–1.20	0.39	1.26–1.30	1.03–1.15	1.10–1.15	0.39–1.30	25.1

Due to the large dispersion of pH values (3.75–6.96) in wild growth sites of *D. superbus*, the concentrations of Ca and Mg also differed significantly—52 and 106 times, respectively, comparing the minimum and maximum values. Nevertheless, the Ca:Mg ratio ranged only from 5.0 (meadow at the Pededze River) to 8.3 (hillock with a clearing at the farmstead ‘Priežulejas’) indicating the presence of similar soil bedrocks in *D. superbus* growth sites *in situ*. Only one lower Ca:Mg ratio (2.9) was found for soil from the edge of the gravel road near the village Silenieki, which indicates on the presence of dolomite impurities.

### Seed viability and germination

**Figure 3.**
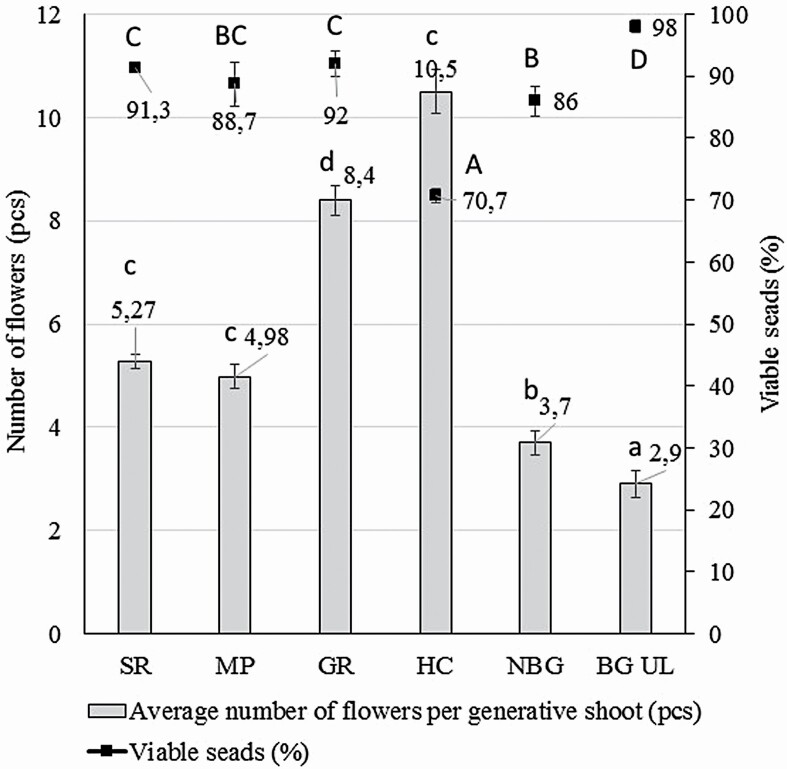
Seed viability and number of flowers per generative shoot of *Dianthus superbus* in 2020 (SR—sand road and the side of the road in pine forest; MP—meadow at the river Pededze; GR—gravel road edge; HC—hillock with clearing at the farmstead ‘Priežulejas’; NBG—National Botanic Garden; BG UL—Botanical Garden of University of Latvia). Means with different letters were significantly different (*t*-test, *P* < 0.05): lower case refers to the number of flowers per generative shoot, upper case refers to seed viability.

Generative capacity—the total number of generative shoots and flowers of *D. superbus* for *in situ* localities—was registered. It was found that none of the localities had <130 generative shoots and 650 flowers. The greatest number of generative shoots and the most abundant flowering was found on the hillock with clearing at the farmstead ‘Priežulejas’ (HC)—in summary 699 generative shoots with 7340 flowers. Seed viability and germination tests performed in our study showed a high percentage of viable seeds of *D. superbus* from all sites *in situ* and *ex situ* (Fig. 3; **see****Supporting Information—Table S1.1**). The highest viability (98 %) was found for seeds sampled in BG LU, where the lowest number of flowers per generative shoot was recorded. It is notable that seeds collected from the *D. superbus* grown in natural conditions of site HC, with significantly higher number of flowers per shoot, exhibited the lowest viability—70.7 %.

To carry out the seed viability test, seeds were soaked in distilled water for 2 days. Within this 2-day period, 41.3 % (HC, *20*) to 84 % (BG LU) of the soaked seeds had already germinated (Table 5).

**Table 5.** Germinationt rate of *D. superbus* seeds within 2 days. *Means with different letters in a column were significantly different (*t*-test, *P* < 0.05, d > c > b > a).

Sampling site	Germinated seeds (%) ± SE
SR—Sand road and the side of the road in pine forest	80.7 ± 0.67c*
MP—Meadow at the Pededze River	74.7 ± 3.53c
GR—Gravel road edge	55.3 ± 2.00b
HC—Hillock with clearing at the farmstead ‘Priežulejas’	41.3 ± 1.15a
NBG—National Botanic Garden	56.0 ± 2.40b
BG LU—Botanical Garden of the University of Latvia	84.0 ± 1.00cd

### In vitro

In 2018, *D. superbus* was successfully introduced *in vitro* and started to be maintained in *in vitro* collection of NBG. Plant tissue culture left for preservation in the *in vitro* collection was alternately kept at a low temperature and in a warm cultivation chamber in line with the methodology. If necessary, it can also serve as a basis for the renewal of outdoor plantings.

### Ex vitro

**Figure 4.**
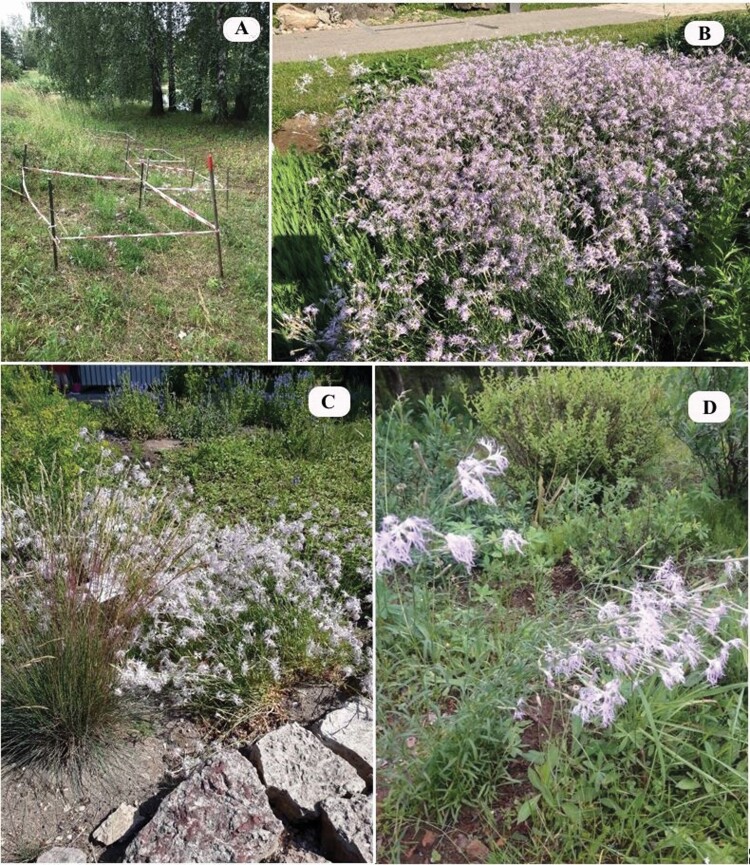
Dianthus *superbus ex vitro* in 2019: (A) pond south slope, semi-natural conditions in NBG (National Botanic Garden); (B) perennial-bed in NBG (photo: Gunta Jakobsone); (C and D) in collection in BG UL (Botanical Garden of University of Latvia) (photo: Ieva Akmane).

**Figure 5.**
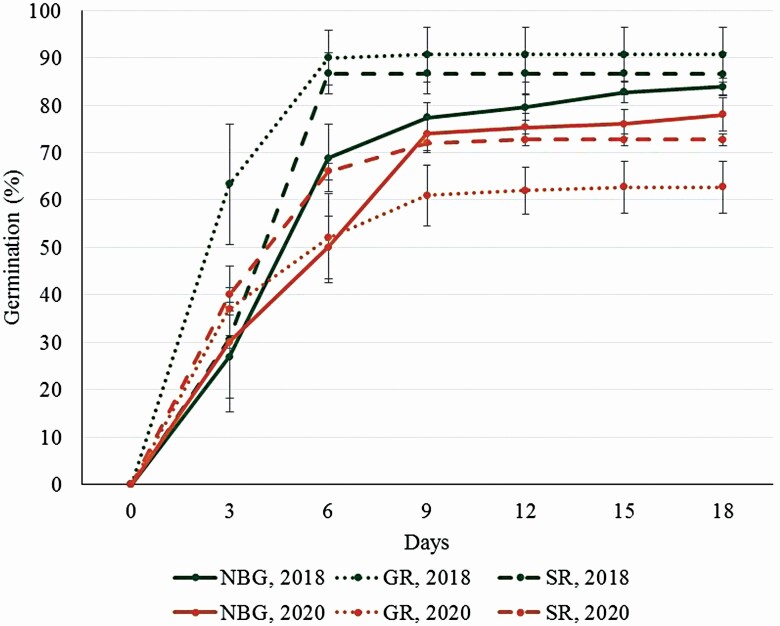
Germination of *Dianthus superbus* seeds: collected in 2017, germinated in spring 2018; collected in 2019, germinated in spring 2020. NBG—National Botanic Garden; GR—gravel road edge; SR—sand road and the side of the road in pine forest.

*Dianthus superbus* in semi-natural conditions in NBG (Fig. 4A) was characterized by the number of flowers per generative shoot and the percentage of germination and viable seeds (Figs 3 and 5; **see****Supporting Information—Table S1.1****and****S1.2**). The results showed high and comparable seed germination and viability, while the number of flowers per generative shoot was lower than under natural conditions of *D. superbus*. The observations made during flowering in 2019 revealed that *D. superbus* in the ornamental bed for perennials in NBG demonstrated high decorativeness when planted closely (Fig. 4B). For *D. superbus* plantings in BG UL (Fig. 4C and D), the light conditions on the shore of the ponds could be described as partial shading, but in the decorative plant bed—as full lighting. In the first year after planting, the largest flowering was found in the site of the ornamental bed, but in the second year, the flowers no longer developed. A more stable situation was found on the shores of ponds, with shoots of *D. superbus* developing in the first year and flowers in the second year. In general, plants grown *ex situ* were qualitatively equivalent to those obtained *in situ.*

## Discussion

### Habitat characteristics

*Dianthus superbus* which is native to most European countries is also an endangered species throughout Europe. Nowadays it is very rarely found as a wild plant in Latvia. Expeditions to update information on 18 previously registered locations revealed only three existing *D. superbus* localities in Latvia with a significant population size (<400 generative shoots) near Lake Lubāns. However, during repeated expeditions, two more sites with flowering plants were successfully found near the above-mentioned locations, increasing the number of deposits to five.

In 2016–20, national monitoring of habitats, plant species and other values of nature called ‘a Nature Census’ was carried out, including nationwide mapping, to gather detailed and complete information on Latvia’s natural capital. Although this project did not include studies on specific plant species, both many rare species and new species were identified. Unfortunately, no new localities of *D. superbus* were found during this survey. This indirectly confirms that *D. superbus* is indeed very rare in Latvia.

A number of literature sources indicated on fens and moderately moist meadows as *D. superbus* habitats (Fleischer and Lindeman 1839; Gavrilova 1999; Kabucis 2003; Kostrakiewicz-Gieralt 2013; Hardion *et al.* 2018). The only place that met this characteristic was micro-reserve Dzirnieki (*DZ*), where *D. superbus* was growing in a meadow with a dense accompanying species cover. In addition, the meadow was regularly mowed low. This generally led to near-disappearance of *D. superbus* in this deposit, as only one specimen was found. This species, which was observed in the lower reaches of the Pededze River even in 2015, was no longer found in 2017 (Jakobsone *et al.* 2020). Here, too, the vegetation cover was probably too high and dense, but *D. superbus* needs enough sunlight, and the growth and development of this species may require a less disruptive root system of other plants. The competition for light and resources in the distribution areas was reported as the main factor determining the growth and development of *Dianthus* species. Decreasing light entering the fen meadows at ground level can inhibit plant growth or even cause mortality (Onete *et al.* 2010; Kostrakiewicz-Gieralt 2014). Studies in Poland conducted in unmanaged *Molinietum caeruleae* meadows situated along the successional gradient have shown that the number of generative shoots, flowers, fruits and seeds of *D. superbus* gradually decreased from places overgrown by shrubs and trees to places with low meadow species. However, significant production of generative structures may not be sufficient to ensure the sustainability of populations in meadows in progressive stages of succession (Kostrakiewicz-Gieralt 2013; Kostrakiewicz-Gieralt 2014). Thus, there is a high risk of extinction of *D. superbus* in wet and humid meadows.

Almost all *D. superbus* localities in Latvia, found in the Lubana wetlands near Silenieki, were on dry sand (forest road) and sand-gravel substrate (road edge, clearing) with accompanying species such as *D. arenarius* and *Thymus serpyllum* etc. Therefore, *D. superbus* habitats in Latvia are similar to Finland, where sandy and gravelly river banks, shore meadows, rocky embankments, dry calcareous grasslands, roadsides are reported as habitat of this species (Lehmuskallio 2014). Obviously, *D. superbus* has a rather wide range of growth conditions.

### Soil agrochemical characteristics

*Dianthus superbus* is generally considered to be a species that prefers fertile, moist but well-drained soil with high organic matter content and a neutral to alkaline soil reaction in the pH range of 6–8 (Dansereau *et al.* 2007; https://pfaf.org 2021). However, some sources suggest that, although preferring alkaline soils, *Dianthus* tolerates low acidity (https://plants.ces.ncsu.edu/plants/dianthus). In Latvia *D. superbus* localities were found mainly on acid soils with pH_KCl_ 3.75–5.27. In contrast, in botanic gardens, vital growth of *D. superbus* was also observed in soils with near-neutral pH level and significantly higher Ca and Mg concentrations (Tables 3 and 4). Therefore, soil reaction for sustainable growth for *D. superbus* could not be considered as a significant limiting factor.

Adequate levels of macronutrients in the soil are an important aspect of ensuring a favourable plant growth condition. From the point of view of plant mineral nutrition, the optimal Ca:Mg ratio in soils using 1 M HCl extraction is 5–8:1 (Rinkis and Nollendorf 1982; Cekstere and Osvalde 2013). Our results revealed that despite the exact soil content of Ca and Mg, their ratio was optimal for the accumulation of these nutrients in the plant. While the levels of P and K in *D. superbus* soils in Latvia were generally comparable to those recommended for crop plants in 1 M HCl extraction, concentrations of N and S can be assessed as very low (Osvalde 2011). However, the relatively high content of soil organic matter (between 3 and 6 %), during mineralization, can provide sufficient amounts of N and S for successful plant growth.

Although relatively wide concentration ranges of mobile forms of micronutrients Fe, Mn, Zn, Cu, Mo and B in soil were found for *D. superbus in situ* and *ex situ* in Latvia, the higher concentrations of Zn, Cu and B were found in botanical gardens. However, considering the low pH of soils from *D. superbus* localities, which promotes the uptake of trace elements, the low status of Cu and B in wild soils may be sufficient to meet plant needs.

Thus, soil agrochemical analyses showed a high probability of successful growth of *D. superbus* in Latvia in soils with a wide nutrient and pH range. In addition, several studies have shown that *D. superbus* is also salt-tolerant (Ma *et al.* 2017) and resistant to heavy metal contamination (Yang *et al.* 2017). Thus, soil chemical content could not be considered as limiting factors for the growth and development of this species. However, optimal nutrient supply would improve the vitality of *D. superbus* in both *ex situ* plantings and wild populations *in situ*.

### Species conservation options

The most effective approach for conservation of endangered plant species can be achieved by combining *in situ* and *ex situ* conservation methods (Miranto *et al.* 2012; Cristea *et al.* 2013). Although *D. superbus* is included in the list of specially protected vascular plants for which micro-reserves shall be established (Regulations of the Cabinet of Ministers of the Republic of Latvia No. 940), the prospect of *in situ* conservation to ensure the protection of this species in Latvia by establishing micro-reserves is limited, as the existing deposits are mainly located in a cemetery and on roadsides. Therefore, *ex situ* conservation of *D. superbus* outside the natural habitats was started by introducing and maintaining *in vitro* collections and field collections in the Latvian Botanic Gardens.

Conservation of *D. superbus* using *in vitro* methods ensures a high rate of propagation and the possibility of obtaining healthy plant material. However, there are potential weaknesses of *in vitro* conservation in relation to vegetative means. For instance, there is the need to develop a propagation protocol for the specific species, the need for a relatively high level of technology and high maintenance costs (Thormann *et al.* 2006). In addition, the most important issue could be the microplant acclimatization *ex vitro* (Muszyńska and Hanus-Fajerska 2017). The simultaneous exposure of plants to many stressors under natural conditions, such as broad-spectrum sunlight, variable temperatures, antagonistic soil microbial communities, can lead to high mortality. On the other hand, the break-up of potential symbiotic interactions could also be a disadvantage of the *in vitro* propagated plants.

Micropropagation and medium-term preservation protocols were developed for *D. superbus* using explants of flower buds, single node stem fragments and sterile sowing of seeds in Murashide and Skoog (1962) medium with various modifications and supplementations (Mikulík 1999; Holobiuc *et al.* 2004–2005). Mikulík (1999) reported the best results in medium without growth regulators, when 86.6 % of rooted shoots and 4.4 axillary shoots per plantlet were obtained. Our study shows that *D. superbus* could be successfully induced and maintained *in vitro* tissue cultures using shoot explants obtained *in situ*. The obtained results revealed that *in vitro* propagation of this species is very simple and fast, and no problems were found for further adaptation of seedlings to *ex situ* conditions. Cuttings from *in vitro* derived seedlings were well rooted in a commercial fertilized sphagnum peat substrate with low microbiological activity. Furthermore, high survival rates were recorded after transplantation under soil conditions. Thus, *in vitro* propagated *D. superbus* was hardly sensitive to soil microbiological composition.

The preservation of biodiversity is closely connected with the task of growing endangered species in botanical gardens for possible reintroduction of these *in situ*. Therefore, after acclimatization, *in vitro* obtained *D. superbus* seedlings were transplanted outdoors in the cultivation sites in the territory of NBG and BG UL. Overall good adaptation and growth success were found in both botanic gardens—the plants bloomed and matured seeds. In general, seed viability and germination are decisive indicators for assessing the reproductive prospects of *in vitro* derived plants. Our results revealed high potential of these living *D. superbus* collections as raw material for reintroduction as a very high percentage of seed viability and germination was found both for *in situ* and *ex situ* growing plants.

New approaches have been proposed in recent decades, integrating *ex situ* and *in situ* conservation for endangered plant species. The *inter situs* approach includes the establishment of species by reintroduction to locations outside the current range—not only in the priority areas within the species past range, but also including locations with some degree of environmental degradation. Such assisted colonization of threatened species also demands agricultural management, invasive species control and plant protection measures (Volis 2017). Restoration of endangered species, combined with the simultaneous restoration of degraded habitat, could be promising nowadays.

Given our data on the width of the species niche in terms of soil conditions (wide nutrient and pH range), *D. superbus* would be suitable for such *inter situs* conservation approach. The first attempts to preserve *D. superbus inter situs* have already taken place in Latvia, as *in vitro* propagated seedlings were planted in Lēdurga Dendrological Park, where they began to successfully grow, bloom and produce seeds.

Due to the ornamental nature of the species and the pleasant aroma of flowers, some places in the urban environment could also serve as alternative habitats for *inter situs* conservation. In this respect, Tallinn, the capital of Estonia, is known to have habitats where protected plant species, including *D. superbus*, thrive (Vacht *et al.* 2018).

In the conditions of sufficient funding for reintroduction projects and national-level support, the integration of *in situ*, *ex situ* and *inter situs* conservation strategies for *D. superbus* in Latvia could be the most optimal. As *D. superbus* is a species that is able to adapt to different soil conditions and is decorative, reintroduction *inter situs* in different environments, including the urban environment, can be considered as a very promising conservation measure.

### Reproduction characteristics and potential

Comparing the generative indicators, a negative correlation was found between the number of flowers per shoot and seed viability (Fig. 3). Thus, the highest number of flowers per generative shoot and the lowest seed viability was found for the site hillock with clearing (*HC*), while the opposite was characteristic for *D. superbus* growing in the botanical garden (BG UL). This could be explained by a lack of resources, as the leaves and roots of the plant were less able to supply seeds with nutrients to shoots that produced 10.5 flowers per shoot than to shoots that produced only 2.9 flowers.

One of the conditions for the spread of the species is the presence of pollinators. The flowers of *D. superbus* are strongly scented, especially during the night, indicating pollination by night-active flower visitors. According to Erhardt’s research in the Swiss Alps (1991) *D. superbus* is adapted to crepuscular and nocturnal hawk-moth and is clearly not pollinated by butterflies, not even diurnal moths. Insect pollinators have not been studied in our work, but a significant number of generative shoots and flowers found both *in situ* and *ex situ* may enhance the chances for nocturnal pollinator visits. The increase in seed production could contribute to the continued survival of the *D. superbus* until overgrowth with other species increases or destructive human activity prevails.

## Conclusions

Expeditions on 18 previously documented locations revealed only three existing *D. superbus* localities in Latvia with a significant number of specimens located in the Latgale region near Silenieki. In 2020, for the first time, two more new *D. superbus* deposits were found in the vicinity of these three approved locations. *In situ* conservation to ensure the protection of *D. superbus* in Latvia by establishing micro-reserves is limited, as the existing deposits are mainly located in a cemetery and on roadsides. Therefore, *ex situ* conservation of *D. superbus* outside natural habitats was carried out by introducing and maintaining *in vitro* collections and field plantings in the National Botanic Garden (NBG) and the Botanical Garden of University of Latvia (BG UL). Our study revealed equivalent quality and high potential of these living *D. superbus* collections as raw material for reintroduction since a very high percentage of seed viability and germination was found both for *in situ* and *ex situ* growing plants. The conservation of this species can also be implemented *inter situs*—in urban areas and dendrological parks by planting *in vitro* propagated plants in perennial ornamental plant beds with the recommendation to use concentrated plantings. The plantings will be ornamental not only for ~1.5 months until a large part of the shoots have stopped flowering, but also after pruning, because, young small shoots with greenish silver leaves are also decorative. The agrochemical results showed that *D. superbus* is a species that is able to adapt to a wide range of soil conditions in terms of nutrient concentrations, soil reaction and organic matter contents. Thus, it can be concluded that there are three main preconditions for the successful growth of *D. superbus* in Latvia—adequate lighting, low overgrowth of other plant species and non-destructive activity of humans.

## Supporting Information

The following additional information is available in the online version of this article—

**Table S1.1**. Data on mean seed viability and number of flowers per generative shoot of *Dianthus superbus* in Latvia, 2020.

**Table S 1.2**. Data on mean germinattion rate of *Dianthus superbus* seeds (%): germinated in spring 2018 (collected in 2017); germinated in spring 2020 (collected in 2019). NBG – National Botanic Garden; GR - gravel road edge; SR - sand road and the roadside in pine forest.

plab051_suppl_Supplementary_Materials

## Data Availability

The data are provided as **Supporting Information**.

## Notes

**Natural History and Conservation. Chief Editor:** F. Xavier Picó
