# Supplementary material for: Dianthus superbus as a critically endangered species in Latvia: evaluation of its growth conditions and conservation possibilities
Source: AoB Plants. 2021 Aug 7;13(5):plab051. doi: 10.1093/aobpla/plab051 (PMC8420110; doi:10.1093/aobpla/plab051)
Supplement: plab051_suppl_Supplementary_Materials [file plab051_suppl_supplementary_materials.docx]

**Supporting Information** for Osvalde et al., “ *Dianthus superbus* L. as a critically endangered species in Latvia: evaluation of its growth conditions and conservation possibilities”

**Supplement 1.**

Table S1.1. Data on mean seed viability and number of flowers per generative shoot of *Dianthus superbus* in Latvia, 2020.

| Localities | Site abbreviation | Mean number of flowers per generative shoot  ± SE | Mean number of viable seeds ( %) ± SE |
| --- | --- | --- | --- |
| Sand road and the roadside in pine forest | SR | 5.27 ± 0.14 | 91.30 ± 0.67 |
| Meadow at the river Pededze | MP | 4.98 ± 0.24 | 88.70 ± 3.53 |
| Gravel road edge | GR | 8.40 ± 0.29 | 92.00 ± 2.00 |
| Hillock with clearing at the farmstead "Priežulejas" | HC | 10.50 ± 0.43 | 70.70 ± 1.15 |
| National Botanic Garden | NBG | 3.70 ±0.24 | 86.00 ± 2.40 |
| Botanical Garden of the University of Latvia | BG UL | 2.90 ± 0.25 | 98.00 ± 1.00 |

Table S 1.2. Data on mean germinattion rate of *Dianthus superbus* seeds (%): germinated in spring 2018 (collected in 2017); germinated in spring 2020 (collected in 2019). NBG – National Botanic Garden; GR - gravel road edge; SR - sand road and the roadside in pine forest.

|  | |  | 2018 |  |  | 2020 |  |
| --- | --- | --- | --- | --- | --- | --- | --- |
| Days | NBG | | GR | SR | NBG | GR | SR |
| 1 | | 0 | 0 | 0 | 0 | 0 | 0 |
| 3 | | 26.90 ± 8.80 | 63.33 ± 12.70 | 30.67 ± 15.40 | 30.00 ±1.30 | 37.00 ± 0.40 | 40.00 ± 1.50 |
| 6 | | 68.80 ± 7.10 | 90.00 ± 5.80 | 86 .70 ± 4.40 | 50.00 ± 6.60 | 52.00 ± 9.40 | 66.00 ± 1.80 |
| 9 | | 77.40 ± 3.20 | 90.67 ± 5.80 | 86.70 ± 4.40 | 74.00 ± 3.50 | 61.00 ± 6.40 | 72.00 ± 2.00 |
| 12 | | 79.60 ± 2.80 | 90.67 ± 5.80 | 86.67 ± 4.40 | 75.3 ± 2.90 | 62.00 ± 5.00 | 72.70 ± 1.30 |
| 15 | | 82.80 ± 2.20 | 90.67 ± 5.80 | 86.64 ± 4.40 | 76.00 ± 3.00 | 62.70 ± 5.50 | 72.70 ± 1.30 |
| 18 | | 83.87 ± 1.90 | 90.67 ± 5.80 | 86.61 ± 4.40 | 78.00 ± 3.50 | 62.70 ± 5.50 | 72.70 ± 1.30 |
